# Development and Validation of Simplified Delirium Prediction Model in Intensive Care Unit

**DOI:** 10.3389/fpsyt.2022.886186

**Published:** 2022-06-29

**Authors:** Min-Kyeong Kim, Jooyoung Oh, Jae-Jin Kim, Jin Young Park

**Affiliations:** ^1^Institute of Behavioral Sciences in Medicine, Yonsei University College of Medicine, Seoul, South Korea; ^2^Department of Psychiatry, Gangnam Severance Hospital, Yonsei University College of Medicine, Seoul, South Korea; ^3^Department of Psychiatry, Yongin Severance Hospital, Yonsei University College of Medicine, Yongin, South Korea; ^4^Center for Digital Health, Yongin Severance Hospital, Yongin, South Korea

**Keywords:** delirium, intensive care unit, prediction model, simplified model, critical care

## Abstract

**Background:**

The intensive care unit (ICU) is where various medical staffs and patients with diverse diseases convene. Regardless of complexity, a delirium prediction model that can be applied conveniently would help manage delirium in the ICU.

**Objective:**

This study aimed to develop and validate a generally applicable delirium prediction model in the ICU based on simple information.

**Methods:**

A retrospective study was conducted at a single hospital. The outcome variable was defined as the occurrence of delirium within 30 days of ICU admission, and the predictors consisted of a 12 simple variables. Two models were developed through logistic regression (LR) and random forest (RF). A model with higher discriminative power based on the area under the receiver operating characteristics curve (AUROC) was selected as the final model in the validation process.

**Results:**

The model was developed using 2,588 observations (training dataset) and validated temporally with 1,109 observations (test dataset) of ICU patients. The top three influential predictors of the LR and RF models were the restraint, hospitalization through emergency room, and drainage tube. The AUROC of the LR model was 0.820 (CI 0.801–0.840) and 0.779 (CI 0.748–0.811) in the training and test datasets, respectively, and that of the RF model was 0.762 (CI 0.732–0.792) and 0.698 (0.659–0.738), respectively. The LR model showed better discriminative power (*z* = 4.826; *P* < 0.001).

**Conclusion:**

The LR model developed with brief variables showed good performance. This simplified prediction model will help screening become more accessible.

## Introduction

Delirium refers to an acute disturbance in awareness and attention caused by various physical problems (1) that affect brain function (2). It results from diverse etiologies, such as systemic illness, substance intoxication or withdrawal, and some of surgeries (2, 3). As delirium occurs under diverse conditions, experts in various fields may often face delirious patients. Each expert uses different terms such as “acute confusional state,” “acute brain failure,” or “intensive care unit (ICU) syndrome” to describe delirium (2, 4). In ICUs, especially in environments where other professionals collaborate, even minor factors such as terminological chaos may hamper effective communication and successful delirium management. Given that ICU delirium demonstrates adverse outcomes, such as increased mortality or length of ICU or hospital stay (5, 6), and occurs frequently (7, 8), this is an issue that should not be overlooked. Therefore, it is essential to establish a system that can detect delirium earlier in ICUs where heterogeneous experts convene.

To date, various prediction models of delirium have been proposed (9–11), and some excellent models have shown high discriminative power only with initial data collected during the first 24 h of admission or at the ICU admission (12, 13). However, most of the existing models include predictors that can be obtained through various tests, such as urea concentration, electrolyte levels, and blood pH (9); therefore, there may be some restrictions on the use of the model if not investigated at a defined time window. In addition, acute physiology and chronic health evaluation II (APACHE II) (14) or mini-mental state examination (15), which are frequently used as predictors, require expert evaluation. While these are undoubtedly significant factors in predicting delirium, obtaining those values without missing data within a specific deadline is not easy. Considering the complexity of the ICU, where various specialists monitor patients with different diseases, a simplified model that is generally applicable for multiple diseases will have clinical significance.

This study aimed to develop and validate a generally applicable delirium prediction model in the ICU with simple, non-missing information. With advances in technology, medical records are stored electronically throughout the hospital stay as electronic health records (EHR) with little omission (16, 17). Simple demographics or primary medical records among vast EHR contain delirium risk factors. Age and drug use are representative risk factors for delirium (18), and these values can be easily extracted from EHRs without missing values.

In this study, we developed a model with improved usability by defining common variables that can be extracted from the EHR of all patients. In addition, we would like to develop a model composed of binary variables where all medical staff can easily use it with a simple selection. To this end, we attempted to develop a more suitable model for data composed of only binary predictors using two analytical methods: logistic regression (LR) and random forest (RF).

## Materials and Methods

### Design and Study Population

This was a retrospective EHR-based study to predict delirium in the ICU. The study was conducted in a 23-bed mixed medical/surgical ICU at a single center (Gangnam Severance Hospital, Yonsei University, Seoul, South Korea). This ICU operates an “ICU Distress and Delirium Management Project” that monitors delirium and distress of patients, and as part of it, psychiatrists assess delirium daily (19). In this study, medical records and demographic information between May 2014 and May 2017 were reviewed. The institutional review board of Gangnam Severance hospital, Yonsei University Health System approved the study procedure.

All patients aged ≥ 20 years who were admitted to the ICU were initially considered for inclusion in the study. Then, patients with following were excluded: (1) coma during the entire ICU stay, (2) length of ICU stay < 24 h, and (3) delirious at ICU admission or within 24 h.

### Outcome Definition and Predictors Selection

The primary outcome variable was the development of delirium during the first 30 days in the ICU. The psychiatrist performed delirium assessment based on the CAM-ICU (20) in the ICU at 10 a.m. when almost all patients could be visited. The evaluation was conducted comprehensively on the progress from the past day and to condition at the time.

Based on reviews (21, 22) and expert opinions in critical care medicine and psychiatry, we established 14 potential predictors that are important factors related to delirium and can be collected with few omissions in most patients in the ICU. All the predictors were set as binary variables for the convenience of response. Predictors were chosen from three domains: patients’ basic information, drug usage, and procedure/intervention application. In the basic information, age, sex, and hospitalization path were used. Age was classified based on whether patients were aged ≥ 65 years, and hospitalization path was classified by whether patients were admitted through the emergency room or outpatient clinic. Risk factors, such as the history of dementia or substance use (23, 24) that may be inaccurate or missing at the beginning of hospitalization, were not used. The conditions related to drug usage and application procedures were based on progress within the first 24 h after ICU admission. We classified categories of the drug as follows: benzodiazepine (midazolam and lorazepam), propofol, dexmedetomidine, opioid analgesics I (morphine, fentanyl, and remifentanil) mainly administrated intravenously, opioid analgesics II (fentanyl transdermal patch, oral tablet containing oxycodone, oral tablet containing tramadol or codeine, and pethidine), which are usually administered other than intravenously, and antipsychotics (haloperidol, risperidone, olanzapine, quetiapine, and aripiprazole). Drug use was investigated regardless of dosage. Finally, five interventions that were essentially identified and recorded during nursing work were used as predictors: vascular catheterization, Foley catheterization, drainage tube, mechanical ventilation, and restraint.

### Statistics

First, the frequency of predictors was explored in the overall data, and predictors that were < 1% were excluded from the analyses of the prediction model. Based on the day of admission, the first 70% were defined as the training dataset and the remaining 30% as the test dataset. The incidence of delirium, average length of ICU stay, mean age and APACHE II score were explored in these three datasets. To find a high-performance model in this data format, we used two methods: standard LR analysis and RF. LR is a traditional method that models the relationship between dependent variable through the combination of independent variables (25). It is a familiar method, and interpretation is straightforward through the odds ratio (OR). RF is an ensemble method used for both classification and regression (26). It has the advantage of good performance and identifying the importance of variables (27–29). These two methods are widely used in developing prediction models.

For the development of the LR model, the relationship between the predictors and delirium was explored using univariate LR, and predictors with *P*-values < 0.2 were chosen as the candidate variables. Then, the model was developed using stepwise multivariate LR based on the Bayesian information criterion. The discrimination power of the LR model was assessed using the area under the receiver operating characteristic curve (AUROC). Calibration (30) was assessed graphically by plotting the observed and predicted probabilities of delirium (31). The RF model was optimized with 3 repeats of fivefold cross-validation with 1,000 trees by tuning hyperparameters. Discrimination power was assessed by AUROC, and the importance of the predictors was explored by calculating the mean decrease in Gini (32). The higher mean decrease in Gini indicates greater importance of the feature in the model. Important predictors of LR and RF models were manually inspected.

The models were validated using the test dataset. The AUROC values of both models were quantified and compared using the DeLong’s test (33). The model with the best performance was finally selected based on AUROC. We explored the sensitivity, specificity, positive likelihood ratio, and negative likelihood ratio of the models with three cutoffs, 10, 20, and 30%, considering the incidence of delirium in the dataset.

Analyses were performed using R statistics version 4.1.2 (34).

## Results

**FIGURE 1:**
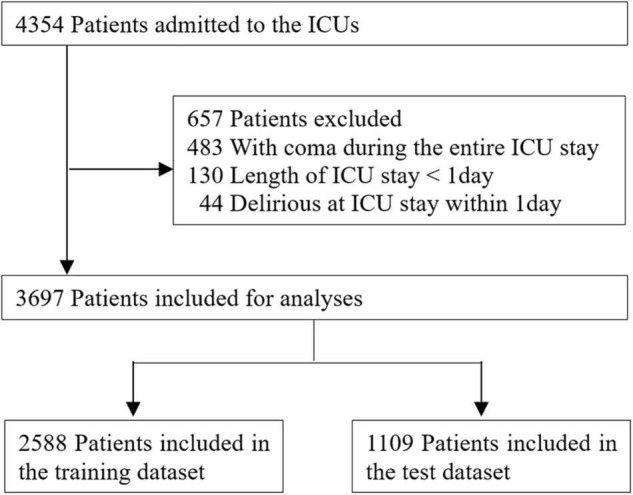
Study flow chart.

Of the total 4,354 adult patients, 3,697 (85%) were included in the study. A total of 657 patients were excluded for the following reasons: 483 patients who were comatose during the ICU stay, 130 patients who had < 24 h of ICU stay, and 44 patients who were in a delirious state on the day of ICU admission (Figure 1). Predictors using propofol (0.16%, 4 patients) and antipsychotics (0.52%, 23 patients) were excluded from further analyses. Among the 3,697 patients, 741 (20.0%) developed delirium, and 2,246 (60.8%) were males. The mean age and mean length of ICU stay of the whole dataset were 63.99 (standard deviation [SD] 15.52) and 5.76 days (*SD* 10.07), respectively. The APACHE II score was obtained from 2,212 observations of the whole dataset, and the mean score was 15.78 (*SD* 7.49). In the training and test datasets, scores were collected from 1,534 and 678 observations, and the average scores were 15.90 (*SD* 7.54) and 15.51 (*SD* 7.38), respectively. The characteristics of each dataset are listed in Table 1.

**TABLE 1:** Patient characteristics.

Variables	Whole dataset (*n* = 3,697)	Train dataset (*n* = 2,588)	Test dataset (*n* = 1,109)
Delirium, *n* (%)	741 (20.04)	517 (19.98)	224 (20.20)
Male/female, *n* (%)	2,246/1,451 (60.8/39.2)	1,567/1,021 (60.5/39.5)	679/430 (61.2/38.8)
Age, mean (*SD*)	63.99 (15.52)	64.21 (15.44)	63.43 (15.71)
Length of ICU stay, in days (*SD*)	5.76 (10.07)	5.85 (10.68)	5.54 (8.48)

### Development of the Logistic Regression and Random Forest Model

The results of the univariate and multivariate LR are shown in Table 2. In univariate regression, all but sex (Coefficient 0.010, *P* = 0.92) and vascular catheterization (Coefficient 0.019, *P* = 0.87) were identified as candidate variables. According to the stepwise multivariate LR, age, hospitalization path, application of restraint, drainage tube, benzodiazepines, and opioid analgesics II remained in the final model (Coefficients were 0.894, 1.249. 1.659, -1.024, 0.815, -0.393, respectively). The LR model was well-calibrated graphically, and the AUROC of the LR model was 0.820 [95% confidence interval (CI) 0.801–0.840]. In the RF model, the number of predictors sampled randomly as candidates at each split was set to three, and the model showed an AUROC of 0.762 (CI 0.732–0.792).

**TABLE 2:** Variables of the delirium prediction model and regression coefficients.

	Univariate analysis		Multivariate analysis	
	Coefficient	*P*-value	Coefficient	*P*-value
Age ≥ 65	0.758	<0.001	0.894	<0.001
Hospitalization path (Emergency room vs. outpatient clinic)	1.517	<0.001	1.249	<0.001
Applying restraint	1.581	<0.001	1.659	<0.001
Applying drainage tube	–1.248	<0.001	–1.024	<0.001
Using benzodiazepines*^a^*	1.553	<0.001	0.815	<0.001
Using opioid analgesics II*^b^*	–1.033	<0.001	–0.393	<0.01
Sex (male vs. female)	0.010	0.92		
Mechanical ventilation	1.272	<0.001		
Applying vascular catheter	0.019	0.87		
Applying Foley catheter	0.359	<0.01		
Using opioid analgesics I*^c^*	1.018	<0.001		
Using dexmedetomidine	0.853	0.03		

*Risk of delirium = 1/(1 + exp – (–3.118 + 0.894 for age ≥ 65 + 1.249 for hospitalization path (emergency room) + 1.659 for applying restraint – 1.024 for applying drainage tube + 0.815 for using benzodiazepines - 0.393 for use of other opioid analgesics)). ^a^Benzodiazepines, including midazolam and lorazepam. ^b^Opioid analgesics II, including fentanyl transdermal patch, oral tablet containing oxycodone, oral tablet containing tramadol or codeine, and pethidine. ^c^Opioid analgesics I, including morphine, fentanyl, and remifentanil.*

**FIGURE 2:**
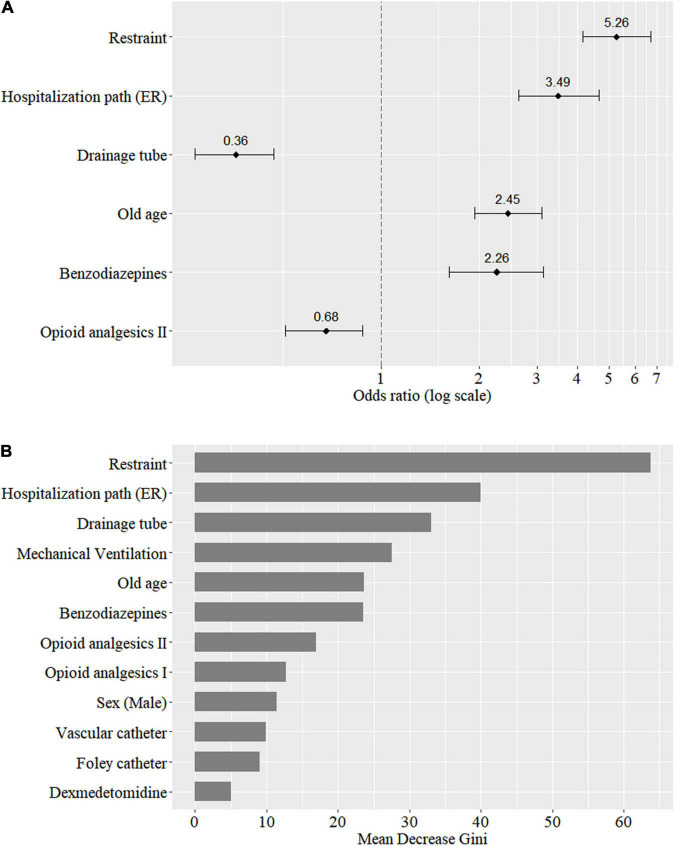
Importance of predictors based on the odds ratio of the logistic regression model **(A)** and the mean decrease in Gini of the random forest model **(B)**.

The OR of the LR model and the mean decrease in the Gini impurity index of the RF model are presented in Figure 2. In the LR model, applying restraint (OR 5.26; CI 4.14–6.69), hospitalization in the emergency room (OR 3.49; CI 2.64–4.64), old age (OR 2.45; CI 1.93–3.11), and benzodiazepine use (OR 2.26; CI 1.62–3.14) showed a positive association, and applying drainage tube (OR 0.36; CI 0.27–0.47) and opioid analgesics II use (OR 0.68; CI 0.51–0.88) showed a negative association with delirium development. In RF model, applying restraint is top-ranking, followed by hospitalization path, applying drainage tube, mechanical ventilation, and old age, which showed mean decrease in Gini of 63.74, 39.92, 33.09, 27.55, and 23.64, respectively. The three upper important features of the RF model were same as the LR model.

### Temporal Validation and Selection of the Final Model

**FIGURE 3:**
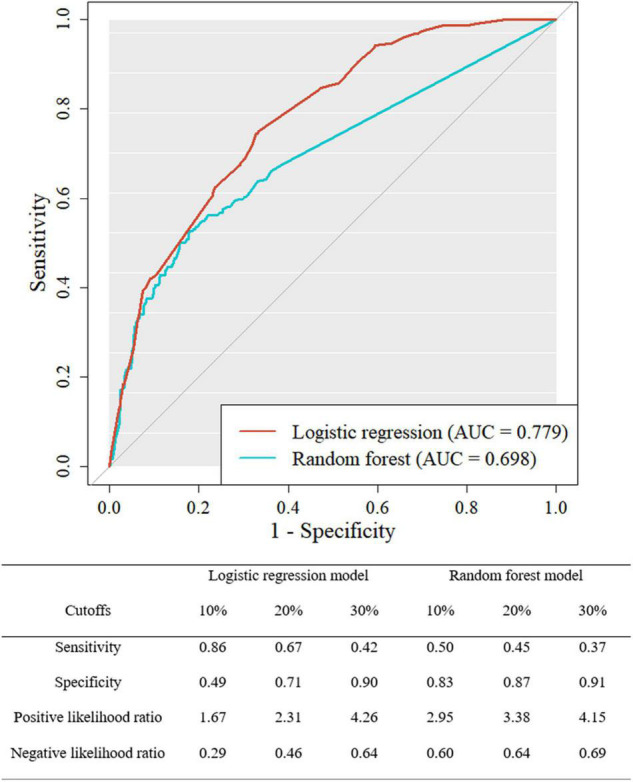
Receiver operating characteristic curve for the logistic regression and random forest model of the test dataset.

ROC curve and the sensitivity, specificity, positive likelihood ratio, and negative likelihood ratio of both models at three cutoffs are presented in Figure 3. The AUROC of the LR and RF models for the test dataset was 0.779 (CI 0.748–0.811) and 0.698 (0.659–0.738). DeLong’s test found that the LR model showed better discriminative power than the RF model (z = 4.826, *P* < 0.001). The final model of this study was selected as the LR model based on the AUROC. The LR model showed the sensitivity of 0.86, 0.67, and 0.42 with the cutoff of 10, 20, and 30%, respectively. The sets of specificity in these cutoffs were 0.49, 0.71, and 0.90. In the RF model, the sensitivity of 0.50, 0.45, and 0.37 and the specificity of 0.83, 0.87, and 0.91 were found at these cutoffs, respectively.

## Discussion

This study aimed to develop an ICU delirium-simplified prediction model using predictors that can be readily measured. Here, the easily measurable predictors have been defined as: (1) a value that could be obtained from almost all patients on their first day at the ICU and (2) a value that would not exert extensive effort to acquire. To find a model that reflects the characteristics of these factors well, we performed two types of analysis: LR and RF. Based on AUROC, the LR model showed better discriminative power compared to the RF model. The LR model, which consisted of six predictors (old age, hospitalization through the emergency room, applying restraint, drainage tube, using benzodiazepines, and some types of opioid analgesics), showed acceptable to excellent discriminative power (25).

The final LR model would maximize the strength of the delirium prediction model. This model can be performed quickly and easily, reducing the burden on staff members working in a high-pressure environment caring for patients with severe diseases and complications. Indeed, six predictors can be found in EHR within just minutes, without particular expertise; such good usability is critical to the success of new initiatives in the ICU (35). Strengths or barriers of applying this model to the clinical environment should be investigated additionally; however, since the model only requires simple data, the introduction process will be relatively straightforward, whether a person calculates the delirium risk directly or develops an automatic calculation system. The main reason for developing delirium prediction models is that while prevention is quite effective, patients with delirium are often underdiagnosed (23, 36). Starting with the advantages of the easy introduction of the system, it is expected to allow the proper allocation of medical and nursing resources and positively affect patient outcomes.

The final simplified model re-confirmed the three risk factors of applying restraint, old age, and benzodiazepine use. The most influential in predicting delirium was the restraint application within 24 h. This result can be thought of in two ways. One is the harmful effects of restraint itself, which limits movement and creates an unfamiliar and frightening environment (37). Many studies have suggested that physical restraint is a representative risk factor for delirium (38, 39). Second is the possibility of restraint as an appropriate intervention. In our hospital, only inevitable patients were selected and applied restraint. A typical case is a patient with a high possibility of action against treatment, and this clinical judgment may be in a similar context to predicting high-risk patients with delirium. Eventually, patients applying restraint should be observed more carefully for the occurrence of delirium. In addition, old age (18), and benzodiazepine use (40, 41) were still identified as solid risk factors for delirium. We found it meaningful to predict delirium by examining the elderly based on the age of 65 and evaluating the use of drugs, not the amount of drugs used.

This model also discovered new meanings for the three variables, hospitalization path, drainage tube, and use of opioid analgesics. The high OR of the “hospitalization path” predictor, defined as hospitalization through the emergency room or outpatient clinic, might be due to the following reasons. First, although additional analysis is needed to confirm the differences in disease severity, patients admitted through the emergency room usually have urgent and severe conditions, and their severity may be related to delirium. Second, the hospitalization path may be related to the circadian rhythm of patients. Circadian fluctuations, such as sleep deprivation, influence the development of delirium (42). Hospitalization through outpatient clinics is usually done during the day. However, hospitalization through the emergency room is likely to occur in the evening or early morning, and these patients may have problems with the circadian rhythm. An interesting result that followed was that the OR of the drainage tube was low. This result was due to a therapeutic effect of the drainage tube, which removes various body fluids and improves wound healing (43). We also considered that the patient’s condition for which surgery was possible might influence the outcome. Further research is required to identify the role of the drainage tube on delirium. Finally, only a subset of opioid analgesics, mainly administered by methods other than IV, was included in the model, and the predictor showed a low OR. Different pharmacokinetics between formulations may have induced distinct associations with the development of delirium (44). It should be noted that proper pain management is essential for delirium management in ICUs (45). Overall, this model was in line with the general guidelines for ICU patients (46, 47).

The final selected LR model showed reasonably good performance with only six elementary information. We believe that the characteristics of our dataset had a significant impact on this performance. First, the predictors would be related to the disease course over a slightly broader time range than the transient state. Blood test results or vital signs are excellent in reflecting the patient’s instantaneous condition. However, as a one-time fragmentary result, these indicators have limitations in explaining “disease progress,” such as improvement or deterioration. Conversely, predictors in our dataset, such as drugs or procedures, were more likely to be repeated or maintained over time (longer than seconds or minutes) and related to the patient’s condition. These relatively stable predictors that reflect the disease course would have a positive effect on performance. Second, the selection of essential variables predicting delirium may also be related to model performance. Delirium is not a disease caused by a few specific factors, but a syndrome of decreased brain functions that various factors can cause (1, 2, 47), and this should be considered when the selecting predictors. It would have been helpful to discuss the essential predictors in the ICU environment with various experts. We confirmed that even if the number or type of variables were simplified, a good performance model could be developed if the meaning of the variables was well-established.

In contrast to the LR model, the RF model showed insufficient performance for delirium prediction. In most cutoffs, the RF model showed low sensitivity and high specificity. However, high sensitivity tests would be preferred considering the adverse outcomes and the effects of early intervention on the delirium course, and in turn, the LR model would be more relevant in the ICU environment. We were able to determine the rationale for the relatively low discriminative power of the RF model in the low-dimensional data characteristics and the difference in model configuration methods. Since the number of predictors was not large, even if the number of cases was large, the advantages of RF might not have been significantly expressed (48). In addition, including some variables of low importance in the model might have degraded the performance. Unlike regression analysis, in which only statistically significant variables were selected, the RF model included all variables, so there was a clear difference in constructing the models. For these reasons, the RF method might have had limitations in deriving the best results from the current data.

This study had several limitations. First, there was no external validation process as this study was performed in a single center. Further validation would be required before implementing the model in clinical practice (49). Second, unlike recommended (50), several variables were not past verified predictors and some were newly defined in this study. Those variables were opioid analgesics I and II, hospitalization path, and drainage tube. Therefore, newly defined variables based on expert opinions and the simplified models developed using such variables will require additional validation processes.

## Conclusion

The goal of this study was to create a delirium prediction model that can be easily applied to anyone in a complex ICU environment. The term “easily” here indicates that it does not require much effort to measure the variables collected with little omission, so that the model can be used at any moment. Between the LR and RF methods, the LR model was selected as the final model with better performance. This simplified model will make it easier for clinicians to try screening, making the preventive intervention of delirium more active.

## Data Availability Statement

The datasets presented in this article are not readily available because it requires institutional permission. Requests to access the datasets should be directed to corresponding author.

## Ethics Statement

The studies involving human participants were reviewed and approved by the Yonsei University Health System, Gangnam Severance Hospital, Institutional Review Board. Written informed consent for participation was not required for this study in accordance with the national legislation and the institutional requirements.

## Author Contributions

M-KK, JO, J-JK, and JP conceived the study and participated in its design. M-KK, JO, and JP contributed to the conduct of the study and acquisition of data, analyzed, and interpreted the data. M-KK and JO drafted the initial manuscript. JP supervised the entire research process and revised the manuscript for publication. All authors take responsibility for data integrity, read, and approved the final version of the manuscript.

## Conflict of Interest

The authors declare that the research was conducted in the absence of any commercial or financial relationships that could be construed as a potential conflict of interest.

## Publisher’s Note

All claims expressed in this article are solely those of the authors and do not necessarily represent those of their affiliated organizations, or those of the publisher, the editors and the reviewers. Any product that may be evaluated in this article, or claim that may be made by its manufacturer, is not guaranteed or endorsed by the publisher.
